# Supplementary material for: Nanoscale Curvature Regulates YAP/TAZ Nuclear Localization Through Nuclear Deformation and Rupture
Source: Adv Sci (Weinh). 2025 Jun 3;12(28):2415029. doi: 10.1002/advs.202415029 (PMC12302533; doi:10.1002/advs.202415029)
Supplement: Supplementary file 1 — Supporting Information [file ADVS-12-2415029-s008.pdf]

ADVANCED SCIENCE
Open Access

Supporting Information

for *Adv. Sci.*, DOI 10.1002/advs.202415029

Nanoscale Curvature Regulates YAP/TAZ Nuclear Localization Through Nuclear Deformation and Rupture

Emmet A. Francis, Einollah Sarikhani, Vrund Patel, Dhivya Pushpa Meganathan, Leah Sadr, Anum Tahir, Zeinab Jahed and Padmini Rangamani**

Nanoscale curvature of the plasma membrane regulates mechanoadaptation through nuclear deformation and rupture, Supporting Information

Emmet A. Francis[1,2,*], Einollah Sarikhani[3,*], Vrund Patel[3], Dhivya Pushpa Meganathan[3], Leah Sadr[3], Anum Tahir [3], Zeinab Jahed[3,**], and Padmini Rangamani[1,2,**]

[1]Department of Pharmacology, University of California San Diego, La Jolla, CA, USA
[2]Department of Mechanical and Aerospace Engineering, University of California San Diego, La Jolla, CA, USA
[3]Aiiso Yufeng Li Family Department of Chemical and Nano Engineering, University of California San Diego, La Jolla, CA, USA
[*]these two authors contributed equally to this work
[**]all correspondence may be addressed to zjahed@ucsd.edu and prangamani@health.ucsd.edu

S1 Mesh generation for cells on nanopillar substrates

Cell geometries on substrates with nanopillars were generated in Gmsh [1]. As a reference geometry, we used a vertically shifted version of the axisymmetric cell shape previously defined [2], for which the plasma membrane surface satisfies:

$$\begin{aligned} \left(1 - \frac{(z-h_{NP})^4}{a+(z-h_{NP})^4}\right)(r^2+(z-h_{NP})^2) + \frac{b(r^2+((z-h_{NP})+c)^2)(z-h_{NP})^4}{d+(z-h_{NP})^4} &= R_c^2 \qquad z \geq h_{NP}, \\ r &= R_c \qquad 0 \leq z < h_{NP}, \end{aligned} \tag{S1}$$

where r and z are cylindrical polar coordinates, R_c is the cell-substrate contact radius, and $a, b, c,$ and d are constants adjusted to conserve cell volume for a given contact area and the spacing, height, and radius of nanopillars. The cytosolic volume was computed as:

$$V_{cyto} = V_{shape}(a,b,c,d,R_c) + \pi R_c^2 h_{NP} - V_{NP}(r_{NP}, p_{NP}, h_{NP}, R_c) - V_{nuc}, \tag{S2}$$

where $V_{shape}(a,b,c,d,R_c)$ is the volume of the shape above the nanopillars. We estimated this volume by computing the volume for a discretized version of the contour; given the list of r, z coordinates over the contour ($\mathbf{r} = [r_1, r_2, ..., r_N], \mathbf{z} = [z_1, z_2, ..., z_N]$), the volume is:

$$\begin{aligned} V_{shape} &= \sum_{n=1}^{N-1} \pi \int_{z_n}^{z_{n+1}} \left(r_n + \frac{r_{n+1}-r_n}{z_{n+1}-z_n}(z-z_n)\right)^2 dz \\ &= \sum_{n=1}^{N-1} \pi (z_{n+1}-z_n)\left(r_n^2 + r_n(r_{n+1}-r_n) + \frac{1}{3}(r_{n+1}-r_n)^2\right), \end{aligned} \tag{S3}$$

$V_{NP}(r_{NP}, p_{NP}, h_{NP}, R_c)$ is the volume excluded by the nanopillars over the contact region (see Figure S4B for annotated volume regions):

$$V_{NP}(r_{NP}, p_{NP}, h_{NP}, R_c) = N_{NP}\pi\Bigg[\underbrace{d_{steric}\left(r_{NP}^2 + r_{NP}d_{steric}\frac{\pi}{2} + 2\frac{d_{steric}^2}{3}\right)}_{\text{volume 1 (top)}} + \underbrace{(r_{NP}+d_{steric})^2(h_{NP}-d_{steric}-d_{curv})}_{\text{volume 2 (middle)}} + \underbrace{d_{curv}\left((r_{NP}+d_{steric}+d_{curv})^2 - (r_{NP}+d_{steric}+d_{curv})d_{curv}\frac{\pi}{2} + 2\frac{d_{curv}^2}{3}\right)}_{\text{volume 3 (bottom)}}\Bigg], \tag{S4}$$

where N_{NP} is the number of nanopillars with which the cell intersects according to its specified contact area. For simplicity, we do not consider any nanopillars that would intersect the cell at the boundary of the contact region; that is, N_{NP} must be a whole number.

To determine the boundary coordinates defined by implicit boundaries in Equation (S1), we solved for r at each z value using `solveset` in Sympy [3]. For a given contact area A_c, constants a, b, c, d were adjusted iteratively until the volume was within 1% of the target volume. Small normal displacements of boundary nodes were then applied to adjust the boundary to within 0.1% of the appropriate volume. The U2OS total cell volume (including the nucleus) was fixed at 4,000 μm^3 assuming the resting cell radius is about 10 μm [4].

In the absence of indentation, the nuclear envelope (NE) forms an oblate spheroid:

$$\left(\frac{r}{a_{nuc}}\right)^2 + \left(\frac{z - z_{nuc}}{b_{nuc}}\right)^2 = 1, \tag{S5}$$

such that the nuclear volume is:

$$V_{nuc} = \frac{4}{3}\pi a_{nuc}^2 b_{nuc}. \tag{S6}$$

The center of the nucleus was placed at $z_{nuc} = h_{NP} - \Delta z_{indent} + 0.2\ \mu\text{m} + b_{nuc}$, where Δz_{indent} is the nuclear deformation in the z-direction at the central nanopillar. In cases where the top of the nucleus was within 0.8 μm of the upper plasma membrane, a_{nuc} and b_{nuc} were adjusted while conserving nuclear volume:

$$b_{nuc,new} = \frac{z_{max} - h_{NP} + \Delta z_{indent} - 1.0}{2}, \tag{S7}$$

$$a_{nuc,new} = \sqrt{\frac{3V_{nuc}}{4\pi b_{nuc,new}}}, \tag{S8}$$

where z_{max} is the z value of the upper plasma membrane at $r = 0$. We constrained the nuclear volume by considering data from Koch et al. [5], in which U2OS cells were confined in microtubes and the dimensions of their nuclei were measured. Approximating the nuclei as ellipsoids, the average volume measured across microtube diameters was 550 μm^3; therefore, we fixed nuclear volume at this value in our simulations. In the cases shown in Fig S2, the nucleus is shifted vertically by setting Δz_{indent} to a negative value. Equations governing the shape of the nucleus indented by nanopillars (that is, positive values of Δz_{indent}) are given in Section S5 below.

All resulting mesh statistics are summarized in Table S1.

Table S1: Mesh statistics.

	Constants (in μm)	Vertices	Cells	Size
	$r_{NP} = 0.1, h_{NP} = 1.0, p_{NP} = 5.0, R_c = 20.25$			
Cyto	$a = 2885, b = 0.4428, c = 24.37, d = 10.47$	14965	67920	4.367×10^2 μm^3
PM		4596	8789	3.560×10^2 μm^2
Nuc	$a_{nuc} = 7.740, b_{nuc} = 2.198, z_{nuc} = 3.398$	2652	8928	6.858×10^1 μm^3
NE		1923	124	5.450×10^1 μm^2
	$r_{NP} = 0.1, h_{NP} = 1.0, p_{NP} = 2.5, R_c = 18.52$			
Cyto	$a = 2588, b = 0.4311, c = 20.18, d = 15.84$	23987	114150	4.364×10^2 μm^3
PM		7472	14341	3.201×10^2 μm^2
Nuc	$a_{nuc} = 6.625,\ b_{nuc} = 3.000,\ z_{nuc} = 4.200$	2508	8253	6.868×10^1 μm^3
NE		1821	3548	4.569×10^1 μm^2
	$r_{NP} = 0.1, h_{NP} = 1.0, p_{NP} = 1.0, R_c = 16.55$			
Cyto	$a = 4268\ , b = 0.4244\ , c = 14.53\ , d = 23.70$	39660	184374	4.366×10^2 μm^3
PM		16960	32946	3.431×10^2 μm^2
Nuc	$a_{nuc} = 6.625\ , b_{nuc} = 3.000\ , z_{nuc} = 4.200$	2735	9066	6.870×10^1 μm^3
NE		1970	3840	4.570×10^1 μm^2
	$r_{NP} = 0.5, h_{NP} = 1.0, p_{NP} = 5.0, R_c = 20.01$			
Cyto	$a = 2815\ , b = 0.4390\ , c = 23.66\ , d = 11.38$	14965	67920	4.367×10^2 μm^3
PM		4596	8789	3.560×10^2 μm^2
Nuc	$a_{nuc} = 7.427\ , b_{nuc} = 2.387\ , z_{nuc} = 3.587$	2652	8928	6.858×10^1 μm^3
NE		1923	3748	5.450×10^1 μm^2
	$r_{NP} = 0.25, h_{NP} = 1.0, p_{NP} = 2.5, R_c = 17.45$			
	0.0 μm nuclear shift			
Cyto	$a = 3383\ , b = 0.4265\ , c = 17.13\ , d = 20.07$	24830	116463	4.362×10^2 μm^3
PM		8234	15928	3.065×10^2 μm^2
Nuc	$a_{nuc} = 6.625\ , b_{nuc} = 3.000\ , z_{nuc} = 4.200$	2559	8439	6.869×10^1 μm^3
NE		1861	3626	4.570×10^1 μm^2
	0.6 μm nuclear shift			
Cyto	$a = 3383\ , b = 0.4265\ , c = 17.13\ , d = 20.07$	24851	116920	4.362×10^2 μm^3
PM		8233	15926	3.065×10^2 μm^2
Nuc	$a_{nuc} = 6.625\ , b_{nuc} = 3.000\ , z_{nuc} = 4.800$	2241	7136	6.864×10^1 μm^3
NE		1687	3287	4.567×10^1 μm^2
	1.0 μm nuclear shift			
Cyto	$a = 3383\ , b = 0.4265\ , c = 17.13\ , d = 20.07$	24902	117519	4.362×10^2 μm^3
PM		8234	15928	3.065×10^2 μm^2
Nuc	$a_{nuc} = 6.625\ , b_{nuc} = 3.000\ , z_{nuc} = 5.200$	2086	6554	6.864×10^1 μm^3
NE		1601	3120	4.567×10^1 μm^2
	1.4 μm nuclear shift			
Cyto	$a = 3383\ , b = 0.4265\ , c = 17.13\ , d = 20.07$	24925	117745	4.362×10^2 μm^3
PM		8234	15928	3.065×10^2 μm^2
Nuc	$a_{nuc} = 6.626\ , b_{nuc} = 2.980\ , z_{nuc} = 5.600$	1983	6246	6.866×10^1 μm^3
NE		1527	2975	4.578×10^1 μm^2

Continued on next page

	Constants (in μm)	Vertices	Cells	Size
	flat, $E_{mod} = 10$ GPa, $R_c = 22.48$			
Cyto	$a = 2590$, $b = 0.4059$, $c = 26.64$, $d = 7.967$	4499	17920	4.355×10^2 μm^3
PM		2298	4404	4.169×10^2 μm^2
Nuc	$a_{nuc} = 6.625$, $b_{nuc} = 3.000$, $z_{nuc} = 3.200$	550	1846	6.868×10^1 μm^3
NE		276	486	4.571×10^1 μm^2
	$r_{NP} = 0.5, h_{NP} = 3.0, p_{NP} = 3.5, R_c = 15.5$			
	$\Delta z_{indent} = 0$ μm			
Cyto	$a = 6110$, $b = 0.5586$, $c = 13.23$, $d = 15.62$	28695	136211	4.391×10^2 μm^3
PM		9188	17770	3.111×10^2 μm^2
Nuc	$a_{nuc} = 7.179$, $b_{nuc} = 2.555$, $z_{nuc} = 5.755$	2749	9340	6.862×10^1 μm^3
NE		1943	3786	4.958×10^1 μm^2
	$\Delta z_{indent} = 0.4$ μm			
Cyto	$a = 6110$, $b = 0.5586$, $c = 13.23$, $d = 15.62$	28585	135643	4.390×10^2 μm^3
PM		9191	17776	3.111×10^2 μm^2
Nuc	$a_{nuc} = 6.920$, $b_{nuc} = 2.755$, $z_{nuc} = 5.555$	2918	10026	6.868×10^1 μm^3
NE		2029	3953	4.781×10^1 μm^2
	$\Delta z_{indent} = 0.8$ μm			
Cyto	$a = 6110$, $b = 0.5586$, $c = 13.23$, $d = 15.62$	28507	134895	4.390×10^2 μm^3
PM		9185	17764	3.111×10^2 μm^2
Nuc	$a_{nuc} = 6.701$, $b_{nuc} = 2.955$, $z_{nuc} = 5.355$	3174	11099	6.871×10^1 μm^3
NE		2149	4187	4.691×10^1 μm^2
	$\Delta z_{indent} = 1.0$ μm			
Cyto	$a = 6110$, $b = 0.5586$, $c = 13.23$, $d = 15.62$	28491	134564	4.390×10^2 μm^3
PM		9185	17764	3.111×10^2 μm^2
Nuc	$a_{nuc} = 6.663$, $b_{nuc} = 3.000$, $z_{nuc} = 5.200$	3302	11597	6.868×10^1 μm^3
NE		2238	4362	4.717×10^1 μm^2
	$\Delta z_{indent} = 1.4$ μm			
Cyto	$a = 6110$, $b = 0.5586$, $c = 13.23$, $d = 15.62$	28367	133232	4.391×10^2 μm^3
PM		9182	17758	3.111×10^2 μm^2
Nuc	$a_{nuc} = 6.698$, $b_{nuc} = 3.000$, $z_{nuc} = 4.800$	3617	12913	6.862×10^1 μm^3
NE		2414	4703	4.886×10^1 μm^2
	$\Delta z_{indent} = 1.8$ μm			
Cyto	$a = 6110$, $b = 0.5586$, $c = 13.23$, $d = 15.62$	28322	132753	4.392×10^2 μm^3
PM		9173	17740	3.111×10^2 μm^2
Nuc	$a_{nuc} = 6.733$, $b_{nuc} = 3.000$, $z_{nuc} = 4.400$	3995	14456	6.852×10^1 μm^3
NE		2640	5143	5.102×10^1 μm^2
	$\Delta z_{indent} = 2.0$ μm			
Cyto	$a = 6110$, $b = 0.5586$, $c = 13.23$, $d = 15.62$	28407	132884	4.393×10^2 μm^3
PM		9182	17758	3.111×10^2 μm^2
Nuc	$a_{nuc} = 6.749$, $b_{nuc} = 3.000$, $z_{nuc} = 4.200$	4210	15284	6.839×10^1 μm^3
NE		2776	5408	5.219×10^1 μm^2

Continued on next page

	Constants (in µm)	Vertices	Cells	Size
	$\Delta z_{indent} = 2.4$ µm			
Cyto	$a = 6110$, $b = 0.5586$, $c = 13.23$, $d = 15.62$	28267	131547	4.396×10^2 µm^3
PM		9172	17738	3.111×10^2 µm^2
Nuc	$a_{nuc} = 6.779$, $b_{nuc} = 3.000$, $z_{nuc} = 3.800$	4538	16656	6.811×10^1 µm^3
NE		2969	5781	5.463×10^1 µm^2
	$\Delta z_{indent} = 2.8$ µm			
Cyto	$a = 6110$, $b = 0.5586$, $c = 13.23$, $d = 15.62$	28216	130846	4.400×10^2 µm^3
PM		9160	17716	3.111×10^2 µm^2
Nuc	$a_{nuc} = 6.805$, $b_{nuc} = 3.000$, $z_{nuc} = 3.400$	4869	17912	6.768×10^1 µm^3
NE		3193	6215	5.710×10^1 µm^2

S2 Equations and parameters for YAP/TAZ mechanotransduction model

The model is summarized in terms of species, reactions, and parameters according to the SMART framework [6]. For clarity, the full system of PDEs is given explicitly at the end of this section.

Table S2: Species in YAP/TAZ mechanotransduction model. All initial conditions and diffusion coefficients are used from [2], except YAP/TAZ diffusion coefficients from [7].

	Compartment	D (µm^2 s^{-1})	Initial Condition
Emod*	PM	0	10 GPa
pFAK	Cyto	10	3.000×10^{-1} µM
RhoA_GDP	Cyto	1	1.000 µM
RhoA_GTP	PM	0.3	3.360×10^1 µm^{-2}
ROCK_A	Cyto	75	0.000 µM
mDia_A	Cyto	1	1.000×10^{-2} µM
Myo_A	Cyto	0.8	1.500 µM
LIMK_A	Cyto	10	1.000×10^{-1} µM
Cofilin_NP	Cyto	10	1.800 µM
FActin	Cyto	0.6	1.790×10^1 µM
GActin	Cyto	13.37	4.824×10^2 µM
LaminA	NE	0.001	0.000 µm^{-2}
NPC_A	NE	0.001	0.000 µm^{-2}
YAPTAZ_free	Cyto	80.0	7.000×10^{-1} µM
YAPTAZ_seq	Cyto	80.0	2.000×10^{-1} µM
YAPTAZ_nuc	Nuc	4.0	7.000×10^{-1} µM

*Emod represents the substrate stiffness and also specifies the location of the substrate ($z = 0$ or surface of nanopillars).

Table S3: Reactions in YAP/TAZ mechanotransduction model.

	Reactants	Products	Equation
a_1	[]	['pFAK']	$cyto_{convert}([FAK_{tot}] - [pFAK]) \left(\frac{\rho_{I,bound}}{\rho_{I,max}}\right) \left(\frac{E_{mod}}{C + E_{mod}} k_{sf} + k_f\right)$ where $\left(\frac{\rho_{I,bound}}{\rho_{I,max}}\right) = \exp\left(-\frac{H_{PM}}{H_0}\right) \mathcal{H}(H_{PM}) + \mathcal{H}(-H_{PM})$ on $\Gamma_{substrate}$, $\mathcal{H}$ is the Heaviside function, and $\Gamma_{substrate}$ is the PM in contact with the substrate.

Continued on next page

	Reactants	Products	Equation
a_2	[‘pFAK’]	[]	$k_{df}[pFAK]$
a_3	[‘RhoA_GDP’]	[‘RhoA_GTP’]	$[RhoA_{GDP}]cyto_{convert}k_{fkrho}(\gamma[pFAK]^5+1)$ $-\frac{[RhoA_{GTP}]k_{drho}}{N_{conv}}$
a_4	[]	[‘ROCK_A’]	$[RhoA_{GTP}]k_{rrho}([ROCK_{tot}]-[ROCK_A])$
a_5	[‘ROCK_A’]	[]	$[ROCK_A]k_{drock}$
b_1	[]	[‘mDia_A’]	$[RhoA_{GTP}]k_{mrho}([mDia_{tot}]-[mDia_A])$
b_2	[‘mDia_A’]	[]	$k_{dmdia}[mDia_A]$
b_3	[]	[‘Myo_A’]	$k_{mr}([Myo_{tot}]-[Myo_A])\cdot$ $\left([ROCK_A]\epsilon\frac{1+\tanh(sc1([ROCK_A]-[ROCK_B]))}{2}+1\right)$ $-[Myo_A]k_{dmy}$
b_4	[]	[‘LIMK_A’]	$k_{lr}([LIMK_{tot}]-[LIMK_A])\cdot$ $\left([ROCK_A]\tau\frac{1+\tanh(sc1([ROCK_A]-[ROCK_B]))}{2}+1\right)$ $-[LIMK_A]k_{dl}$
b_5	[]	[‘Cofilin_NP’]	$k_{turnover}([Cofilin_{tot}]-[Cofilin_{NP}])$ $-\frac{[Cofilin_{NP}][LIMK_A]k_{catCof}}{Cofilin_{NP}+k_{mCof}}$
b_6	[‘GActin’]	[‘FActin’]	$[GActin]k_{ra}\cdot$ $\left(\alpha_m[mDia_A]\frac{1+\tanh(sc1([mDia_A]-[mDia_B]))}{2}+1\right)$ $-[FActin]([Cofilin_{NP}]k_{fc1}+k_{dep})$
b_7	[‘GActin’]	[‘FActin’]	$k_{N\text{-}WASP}\exp\left(\frac{H_{PM}}{H_{N\text{-}WASP}}\right)[GActin]$
c_1	[‘YAPTAZ_seq’]	[‘YAPTAZ_free’]	$[YAPTAZ_{seq}]([FActin][Myo_A]k_{CY}+k_{CN})$ $-[YAPTAZ_{free}]k_{NC}$
c_3	[]	[‘LaminA’]	$\frac{[FActin]^{2.6}k_{fl}p([LaminA_{tot}]-[LaminA])}{C_{LaminA}+[FActin]^{2.6}p}-[LaminA]k_{rl}$
c_4	[]	[‘NPC_A’]	$[FActin][LaminA][Myo_A]k_{fNPC}([NPC_{tot}]-[NPC_A])$ $-[NPC_A]k_{rNPC}$

Continued on next page

	Reactants	Products	Equation
c_5	['YAPTAZ_free']	['YAPTAZ_nuc']	$[YAPTAZ_{free}]([NPC_A]k_{in} + k_{inb})\exp\left(\frac{\alpha - 1}{\alpha_0}\right)$ $-[YAPTAZ_{nuc}]k_{out}$
c_6	['YAPTAZ_free']	['YAPTAZ_nuc']	$p_{NER}k_{rupture}([YAPTAZ_{free}] - \frac{[YAPTAZ_{nuc}]}{\upsilon})$

Table S4: Parameters in YAP/TAZ mechanotransduction model. Values were adopted directly from those reported in [2] unless otherwise noted.

	Value	Description
FAK_{tot}	$1.000\,\mu\text{M}$	Total cytosolic FAK
k_f	$1.500 \times 10^{-2}\,\text{s}^{-1}$	rate constant for baseline FAK phosphorylation
k_{sf}	$3.790 \times 10^{-1}\,\text{s}^{-1}$	substrate-stiffness-dependent FAK phosphorylation rate constant
E_{mod}	$1 \times 10^{7}\,\text{kPa}$	substrate stiffness
C	$3.250\,\text{kPa}$	critical substrate stiffness
$cyto_{convert}$	$1.825\,\mu\text{m}$	vol/SA ratio
N_{conv}	$6.022 \times 10^{2}\,\mu\text{m}^{-3}\,\mu\text{M}^{-1}$	unit conversation factor
H_{PM}	Equation (S36) (μm^{-1})	plasma membrane mean curvature
$H_{0,FAK}$	varied (μm^{-1})	curvature sensitivity of FAK phosphorylation
k_{df}	$3.500 \times 10^{-2}\,\text{s}^{-1}$	FAK dephosphorylation rate constant
k_{fkrho}	$1.680 \times 10^{-2}\,\text{s}^{-1}$	RhoA activation rate constant
γ	$7.756 \times 10^{1}\,\mu\text{M}^{-5}$	Scaling factor
k_{drho}	$6.250 \times 10^{-1}\,\text{s}^{-1}$	RhoA deactivation rate constant
k_{rrho}	$6.480 \times 10^{-1}\,\mu\text{M}^{-1}\,\text{s}^{-1}$	ROCK activation rate constant
$ROCK_{tot}$	$1.000\,\mu\text{M}$	Total cytosolic ROCK
k_{drock}	$8.000 \times 10^{-1}\,\text{s}^{-1}$	ROCK deactivation rate constant
k_{mrho}	$2.000 \times 10^{-3}\,\mu\text{M}^{-1}\,\text{s}^{-1}$	mDia activation rate constant
$mDia_{tot}$	$8.000 \times 10^{-1}\,\mu\text{M}$	Total cytosolic mDia
k_{dmdia}	$5.000 \times 10^{-3}\,\text{s}^{-1}$	mDia deactivation rate constant
Myo_{tot}	$5.000\,\mu\text{M}$	Total cytosolic myosin
k_{mr}	$3.000 \times 10^{-2}\,\text{s}^{-1}$	Myosin activation rate constant
$ROCK_B$	$3.000 \times 10^{-1}\,\mu\text{M}$	Critical activated ROCK concentration
ϵ	$3.600 \times 10^{1}\,\mu\text{M}^{-1}$	Scaling factor
$sc1$	$2.000 \times 10^{1}\,\mu\text{M}^{-1}$	Scaling factor
k_{dmy}	$6.700 \times 10^{-2}\,\text{s}^{-1}$	Myosin deactivation rate constant
$LIMK_{tot}$	$2.000\,\mu\text{M}$	Total cytosolic LIMK
k_{lr}	$7.000 \times 10^{-2}\,\text{s}^{-1}$	LIMK activation rate constant
τ	$5.549 \times 10^{1}\,\mu\text{M}^{-1}$	Scaling factor
k_{dl}	$2.000\,\text{s}^{-1}$	LIMK deactivation rate constant
$Cofilin_{tot}$	$2.000\,\mu\text{M}$	Total cytosolic cofilin
$k_{turnover}$	$4.000 \times 10^{-2}\,\text{s}^{-1}$	Cofilin dephosphorylation rate constant
k_{catCof}	$3.400 \times 10^{-1}\,\text{s}^{-1}$	Cofilin phosphorylation rate constant
k_{mCof}	$4.000\,\mu\text{M}$	Critical cofilin concentration
k_{ra}	$4.000 \times 10^{-1}\,\text{s}^{-1}$	Actin polymerization rate constant
α_m	$5.000 \times 10^{1}\,\mu\text{M}^{-1}$	Scaling factor
$mDia_B$	$1.650 \times 10^{-1}\,\mu\text{M}$	Critical activated mDia concentration
k_{dep}	$3.500\,\text{s}^{-1}$	Actin depolymerization rate constant
k_{fc1}	$4.000\,\mu\text{M}^{-1}\,\text{s}^{-1}$	Cofilin-mediated actin depolymerization rate constant
$k_{N\text{-}WASP}$	$1.000 \times 10^{-2}\,\mu\text{m}\,\text{s}^{-1}$	N-WASP-mediated actin polymerization rate constant (see main text)

Continued on next page

	Value	Description
$H_{N\text{-}WASP}$	$2.000\,\mu m^{-1}$	N-WASP curvature sensitivity (see main text and [8, 9])
k_{CN}	$5.600 \times 10^{-1}\,s^{-1}$	Baseline YAP/TAZ desequestration rate constant
k_{CY}	$7.600 \times 10^{-4}\,\mu M^{-2}\,s^{-1}$	stress-fiber-mediated YAP/TAZ de-sequestration rate constant
k_{NC}	$1.400 \times 10^{-1}\,s^{-1}$	YAP/TAZ sequestration rate constant
$LaminA_{tot}$	$3.500 \times 10^{3}\,\mu m^{-2}$	Total lamin A in NE
k_{fl}	$4.600 \times 10^{-1}\,s^{-1}$	Lamin A dephosphorylation rate constant
p	$9.000 \times 10^{-6}\,kPa\,\mu M^{-2.6}$	Scaling factor for cell stiffness
C_{LaminA}	$1.000 \times 10^{2}\,kPa$	Critical cell stiffness for lamin A dephosphorylation
k_{rl}	$1.000 \times 10^{-3}\,s^{-1}$	Lamin A phosphorylation rate constant
NPC_{tot}	$6.500\,\mu m^{-2}$	Total NPC density in NE
k_{fNPC}	$2.800 \times 10^{-7}\,\mu m^{2}\,\mu M^{-2}\,s^{-1}$	NPC opening rate constant
k_{rNPC}	$8.700\,s^{-1}$	NPC closing rate constant
k_{inb}	$1.000\,\mu m^{-2}\,\mu M^{-1}\,s^{-1}$	NPC-independent YAP/TAZ nuclear import rate
k_{in}	$1.000 \times 10^{1}\,\mu M^{-1}\,s^{-1}$	NPC-dependent YAP/TAZ nuclear import rate
k_{out}	$1.000\,\mu m^{-2}\,\mu M^{-1}\,s^{-1}$	YAP/TAZ nuclear export rate
α_{NE}	Equation (S47) (unitless)	stretch ratio of NE
$\alpha_{NE,0}$	varied (unitless)	NPC sensitivity to stretch
υ	varied (unitless)	ratio of import to export through rupture-induced pore
$k_{rupture}$ *	$1.000 \times 10^{5}\,s^{-1}\,\mu M^{-1}\,\mu m^{-2}$	rate of export through rupture-induced pore
p_{NER}	$\left(1 - \exp\left(-\frac{t}{t_0}\right)\right) \cdot \exp\left(-\frac{x^2+y^2+(z-z_{NE})^2}{\sigma_{pore}^2}\right)$	relative permeability of rupture-induced pore
t_0	$1.000\,s$	time constant for pore formation
z_{NE}	$h_{NP} + 0.2\,\mu m$	z-value of NE at which rupture-induced pore forms
σ_{pore}	varied (μm)	effective radius of rupture-induced pore

* Order of magnitude for $k_{rupture}$ is estimated assuming diffusive transport; i.e., $k_{rupture} = N_{convert}\frac{D_{Y,NER}}{d_{NE}}$, where $D_{Y,NER}$ is the effective diffusion coefficient for transport through the NER region (assumed to be between YAP/TAZ diffusion coefficients in the cytosol and nucleus, at about 10 μm ^{2}/s) and d_{NE} is the thickness of the NE (about 50 nm).

The best-fit value of H_0 was determined by assessing the normalized sum of square errors ($NSSE$) between experimental data from [10] and our model over the 5 nanopillar substrates considered here:

$$NSSE = \sum_{k=1}^{5} \left(\frac{1}{SEM_{exp,k}}\right)^2 \left[\left(\frac{Y_{nuc}}{Y_{cyto}}\right)_{exp,k} - \left(\frac{Y_{nuc}}{Y_{cyto}}\right)_{sim,k}\right]^2, \tag{S9}$$

where $SEM_{exp,k}$ is the standard error of the mean associated with the YAP/TAZ N/C measured on substrate k, and $\left(\frac{Y_{nuc}}{Y_{cyto}}\right)_{exp,k}$ and $\left(\frac{Y_{nuc}}{Y_{cyto}}\right)_{sim,k}$ are the mean experimental YAP/TAZ N/C and the spatially averaged YAP/TAZ N/C for substrate k, respectively. The $NSSE$ is plotted as a function of H_0 for two different values of $k_{N\text{-}WASP}$ in Fig S1B.

Given the equations, parameters, and variables defined in the above equations, we can write out the full system of partial differential equations that are solved in SMART. Volume domains are written as Ω (i.e., Ω_{cyto} and Ω_{nuc}) and surface domains are written as Γ (i.e., Γ_{PM} and Ω_{NE}). ∇_S^2 denotes the surface Laplacian operator.

$$\begin{aligned} \frac{\partial [pFAK]}{\partial t} &= D_{FAK}\nabla^2[pFAK] - a_2 \quad \text{in } \Omega_{cyto} \\ -D_{FAK}\mathbf{n}_{PM} \cdot \nabla[pFAK] &= a_1 \quad \text{on } \Gamma_{PM} \\ D_{FAK}\mathbf{n}_{NE} \cdot \nabla[pFAK] &= 0 \quad \text{on } \Gamma_{NE} \end{aligned} \tag{S10}$$

$$\frac{\partial[RhoA_{GTP}]}{\partial t} = D_{RhoA_{GTP}} \nabla_S^2 [RhoA_{GTP}] + N_{conv} a_3 \quad \text{on } \Gamma_{PM} \tag{S11}$$

$$\begin{aligned} \frac{\partial[RhoA_{GDP}]}{\partial t} &= D_{RhoA_{GDP}} \nabla^2 [RhoA_{GDP}] \quad \text{in } \Omega_{cyto} \\ -D_{RhoA_{GDP}} \mathbf{n}_{PM} \cdot \nabla [RhoA_{GDP}] &= -a_3 \quad \text{on } \Gamma_{PM} \\ D_{RhoA_{GDP}} \mathbf{n}_{NE} \cdot \nabla [RhoA_{GDP}] &= 0 \quad \text{on } \Gamma_{NE} \end{aligned} \tag{S12}$$

$$\begin{aligned} \frac{\partial[ROCK_A]}{\partial t} &= D_{ROCK} \nabla^2 [ROCK_A] - a_5 \quad \text{in } \Omega_{cyto} \\ -D_{ROCK} \mathbf{n}_{PM} \cdot \nabla [ROCK_A] &= a_4 \quad \text{on } \Gamma_{PM} \\ D_{ROCK} \mathbf{n}_{NE} \cdot \nabla [ROCK_A] &= 0 \quad \text{on } \Gamma_{NE} \end{aligned} \tag{S13}$$

$$\begin{aligned} \frac{\partial[mDia_A]}{\partial t} &= D_{mDia} \nabla^2 [mDia_A] - b_2 \quad \text{in } \Omega_{cyto} \\ -D_{mDia} \mathbf{n}_{PM} \cdot \nabla [mDia_A] &= b_1 \quad \text{on } \Gamma_{PM} \\ D_{mDia} \mathbf{n}_{NE} \cdot \nabla [mDia_A] &= 0 \quad \text{on } \Gamma_{NE} \end{aligned} \tag{S14}$$

$$\begin{aligned} \frac{\partial[Myo_A]}{\partial t} &= D_{Myo} \nabla^2 [Myo_A] + b_3 \quad \text{in } \Omega_{cyto} \\ -D_{Myo} \mathbf{n}_{PM} \cdot \nabla [Myo_A] &= 0 \quad \text{on } \Gamma_{PM} \\ D_{Myo} \mathbf{n}_{NE} \cdot \nabla [Myo_A] &= 0 \quad \text{on } \Gamma_{NE} \end{aligned} \tag{S15}$$

$$\begin{aligned} \frac{\partial[LIMK_A]}{\partial t} &= D_{LIMK} \nabla^2 [LIMK_A] + b_4 \quad \text{in } \Omega_{cyto} \\ -D_{LIMK} \mathbf{n}_{PM} \cdot \nabla [LIMK_A] &= 0 \quad \text{on } \Gamma_{PM} \\ D_{LIMK} \mathbf{n}_{NE} \cdot \nabla [LIMK_A] &= 0 \quad \text{on } \Gamma_{NE} \end{aligned} \tag{S16}$$

$$\begin{aligned} \frac{\partial[Cofilin_{NP}]}{\partial t} &= D_{Cofilin} \nabla^2 [Cofilin_{NP}] + b_5 \quad \text{in } \Omega_{cyto} \\ -D_{Cofilin} \mathbf{n}_{PM} \cdot \nabla [Cofilin_{NP}] &= 0 \quad \text{on } \Gamma_{PM} \\ D_{Cofilin} \mathbf{n}_{NE} \cdot \nabla [Cofilin_{NP}] &= 0 \quad \text{on } \Gamma_{NE} \end{aligned} \tag{S17}$$

$$\begin{aligned} \frac{\partial[FActin]}{\partial t} &= D_{FActin} \nabla^2 [FActin] + b_6 \quad \text{in } \Omega_{cyto} \\ -D_{FActin} \mathbf{n}_{PM} \cdot \nabla [FActin] &= b_7 \quad \text{on } \Gamma_{PM} \\ D_{FActin} \mathbf{n}_{NE} \cdot \nabla [FActin] &= 0 \quad \text{on } \Gamma_{NE} \end{aligned} \tag{S18}$$

(S19)

$$\begin{aligned}\frac{\partial [YAPTAZ_{seq}]}{\partial t} &= D_{YAPTAZ,cyto}\nabla^2[YAPTAZ_{seq}] - c_1 \quad \text{in } \Omega_{cyto} \\ -D_{YAPTAZ,cyto}\mathbf{n}_{PM}\cdot\nabla[YAPTAZ_{seq}] &= 0 \quad \text{on } \Gamma_{PM} \\ D_{YAPTAZ,cyto}\mathbf{n}_{NE}\cdot\nabla[YAPTAZ_{seq}] &= 0 \quad \text{on } \Gamma_{NE}\end{aligned} \tag{S20}$$

$$\begin{aligned}\frac{\partial [YAPTAZ_{free}]}{\partial t} &= D_{YAPTAZ,cyto}\nabla^2[YAPTAZ_{free}] + c_1 \quad \text{in } \Omega_{cyto} \\ -D_{YAPTAZ,cyto}\mathbf{n}_{PM}\cdot\nabla[YAPTAZ_{free}] &= 0 \quad \text{on } \Gamma_{PM} \\ D_{YAPTAZ,cyto}\mathbf{n}_{NE}\cdot\nabla[YAPTAZ_{free}] &= -\frac{c_5 + c_6}{N_{conv}} \quad \text{on } \Gamma_{NE}\end{aligned} \tag{S21}$$

$$\frac{\partial [LaminA]}{\partial t} = D_{LaminA}\nabla_S^2[LaminA] + c_3 \quad \text{on } \Gamma_{NE} \tag{S22}$$

$$\frac{\partial [NPC_A]}{\partial t} = D_{LaminA}\nabla_S^2[NPC_A] + c_4 \quad \text{on } \Gamma_{NE} \tag{S23}$$

$$\begin{aligned}\frac{\partial [YAPTAZ_{nuc}]}{\partial t} &= D_{YAPTAZ,nuc}\nabla^2[YAPTAZ_{nuc}] \quad \text{in } \Omega_{cyto} \\ -D_{YAPTAZ,nuc}\mathbf{n}_{NE}\cdot\nabla[YAPTAZ_{nuc}] &= \frac{c_5 + c_6}{N_{conv}} \quad \text{on } \Gamma_{NE}\end{aligned} \tag{S24}$$

S3 Well-mixed approximation for YAP/TAZ N/C

Here, we assume that YAP/TAZ is well-mixed within each compartment (the cytosol and the nucleus), but other variables may still exhibit some spatial dependence. In this case, dynamics governing YAP/TAZ nuclear translocation is governed by two ODEs. Writing concentrations of free cytosolic YAP/TAZ, sequestered cytosolic YAP/TAZ, and nuclear YAP/TAZ as Y_{free}, Y_{seq}, and Y_{nuc}, respectively, the volume of the nucleus as vol_{nuc}, and the volume of the cytosol as vol_{cyto}:

$$\frac{dY_{free}}{dt} =(k_{CN} + \bar{k}_{CY})Y_{seq} - k_{NC}Y_{free} + \frac{1}{N_{conv}vol_{cyto}}\left(k_{out,tot}Y_{nuc} - k_{in,tot}Y_{free}\right) \tag{S25}$$

$$\frac{dY_{nuc}}{dt} =\frac{1}{N_{conv}vol_{nuc}}\left(k_{in,tot}Y_{free} - k_{out,tot}Y_{nuc}\right) \tag{S26}$$

$$\begin{aligned}&\text{where} \quad Y_{seq} = \frac{Y_{tot} - N_{conv}(Y_{nuc}vol_{nuc} + Y_{free}vol_{cyto})}{N_{conv}vol_{cyto}}, \\ &k_{in,tot} = \bar{k}_{in,NPC} + p_{NER}k_{rupture}SA_{NER}, \\ &\text{and} \quad k_{out,tot} = k_{out,NPC}SA_{nuc} + p_{NER}\frac{k_{rupture}}{v}SA_{NER}\end{aligned}$$

$\bar{k}_{in,NPC}$ is defined to account for spatially dependent stretch-sensitive entry:

$$\bar{k}_{in,NPC} = \int_{\Gamma_{NE}} \left(k_{inb} + k_{in}[NPC_A]\right)\exp\left(\frac{\alpha - 1}{\alpha_0}\right)d\Gamma, \tag{S27}$$

and $\bar{k}_{CY}$ is defined to account for actomyosin activity across the entire cell:

$$\bar{k}_{CY} = \frac{1}{vol_{cyto}} \int_{\Omega_{cyto}} k_{CY}[FActin][Myo_A]d\Omega. \tag{S28}$$

Assuming the rupture-induced pore is located over a flat patch of membrane directly above the central nanopillar, the effective surface area occupied by the pore upon completion of its formation is:

$$SA_{NER} = \int_{-\infty}^{\infty} \int_{-\infty}^{\infty} \exp\left(-\frac{x^2+y^2}{\sigma_{pore}^2}\right) dxdy = \pi\sigma_{pore}^2. \tag{S29}$$

The resulting steady-state YAP/TAZ N/C is given by:

$$\begin{aligned} \frac{Y_{nuc}}{Y_{free}+Y_{seq}} &= \frac{\phi_{free}(\bar{k}_{in,NPC} + k_{rupture}SA_{NER})}{k_{out,NPC}SA_{nuc} + \frac{k_{rupture}}{\upsilon}SA_{NER}}, \\ \text{where} \quad \phi_{free} &= \frac{k_{CN}+\bar{k}_{CY}}{k_{NC}+k_{CN}+\bar{k}_{CY}}. \end{aligned} \tag{S30}$$

We consider a couple specialized cases presented in the main text. In these examples, the NE is split into two regions - one uniformly stretched with associated area dilation α and activated NPC density $[NPC_A]_{def}$ and the other remaining undeformed ($\alpha = 1$ and $[NPC_A] = [NPC_A]_{undef}$). This differs from the non-uniform stretch introduced in Section S5, but introduces a simplified form of Equation (S27):

$$\bar{k}_{in,NPC} = (k_{inb} + k_{in}[NPC_A]_{def}) \exp\left(\frac{\alpha-1}{\alpha_0}\right) SA_{def} + (k_{inb} + k_{in}[NPC_A]_{undef})\, SA_{undef}, \tag{S31}$$

When $k_{rupture} = 0$ (no NER), Equation (S30) then simplifies to the form in the main text (Eq 5). For small deformations over a region of the reference geometry with surface area SA_0, $SA_{def} \approx \alpha SA_0$. For illustration, we plot the predictions of Equation (S31) as a function of α (all other parameters fixed) in Figure S3B.

When $k_{rupture} \neq 0$, we obtain the remaining case in Eq 10 of the main text. For illustration, we then plot the predictions of Equation (S31) as a function of $k_{rupture}SA_{NER}$ (all other parameters fixed) in Figure S3C.

S4 Plasma membrane curvature calculations

Here, we describe our strategy to define plasma membrane shape around nanopillars. Rather than derive membrane morphology from first principles (e.g., energy minimization), we describe cell shape using empirical relationships that match well with cell morphology in experimental images (cf. Fig 3). The curvature of the plasma membrane can then be analytically expressed in the region surrounding each nanopillar.

The membrane shape around each nanopillar is assumed to be axisymmetric with respect to the central axis of the pillar, with a cross-sectional profile composed of lines and circular arcs (Figure S4A-B) We assume that the membrane is attached to the nanopillar surface via adhesive interactions, fixed at a minimum distance of $d_{steric} =$ 50 nm from the surface due to steric repulsion. Furthermore, the membrane curves to meet the lower surface with a characteristic radius, $d_{curv} = 200$ nm, as depicted in Figure S4A.

The segments of this geometry are readily parameterized in terms of arc length s in the r_{local}, z plane, where r_{local} is the radial coordinate defined with the origin at the center of a given nanopillar located at the point (x_{NP}, y_{NP}). That is, $r_{local} = \sqrt{(x-x_{NP})^2 + (y-y_{NP})^2}$, where x and y are the global Cartesian coordinates (all coordinates defined in Figure S4A-B). For a nanopillar of radius r_{NP} and height h_{NP}:

$$r_{local} = \begin{cases} s & 0 \le s < s_1 \\ r_{NP} + d_{steric}\sin\phi & s_1 \le s < s_2 \\ r_{NP} + d_{steric} & s_2 \le s < s_3 \\ r_{NP} + d_{steric} + d_{curv}(1-\sin\phi) & s_3 \le s < s_4 \\ r_{NP} + d_{steric} + d_{curv} + s - s_4 & s \ge s_4 \end{cases}, \tag{S32}$$

and

$$z = \begin{cases} h_{NP} & 0 \le s < s_1 \\ h_{NP} + d_{steric}(\cos\phi - 1) & s_1 \le s < s_2 \\ h_{NP} - d_{steric} - (s - s_2) & s_2 \le s < s_3 \\ d_{curv}(1-\cos\phi) & s_3 \le s < s_4 \\ 0 & s \ge s_4 \end{cases}, \tag{S33}$$

The arc length values s_1, s_2, s_3, and s_4 are shown in Figure S4B and can be expressed in terms of the defined geometric parameters:

$$\begin{aligned} s_1 &= r_{NP} \\ s_2 &= s_1 + \frac{\pi d_{steric}}{2} \\ s_3 &= s_2 + h_{NP} - d_{steric} - d_{curv} \\ s_4 &= s_3 + \frac{\pi d_{curv}}{2} \end{aligned} \tag{S34}$$

ϕ is the polar angle measured between the normal vector $\mathbf{n}$ (Fig S4A) and the z-axis:

$$\phi = \begin{cases} 0 & 0 \le s < s_1 \\ \frac{s - s_1}{d_{steric}} & s_1 \le s < s_2 \\ \frac{\pi}{2} & s_2 \le s < s_3 \\ \frac{s_4 - s}{d_{curv}} & s_3 \le s < s_4 \\ 0 & s \ge s_4 \end{cases}, \tag{S35}$$

Given the axisymmetry of the geometry, curvature can be readily calculated using the following relationship:

$$H = \frac{1}{2}\left(\frac{\partial\phi}{\partial s} + \frac{\sin\phi}{r_{local}}\right) = \begin{cases} 0 & 0 \le s < s_1 \\ \frac{1}{2d_{steric}}\left(1 + \frac{r_{local} - r_{NP}}{r_{local}}\right) & s_1 \le s < s_2 \\ \frac{1}{2(r_{NP} + d_{steric})} & s_2 \le s < s_3 \\ -\frac{1}{2d_{curv}}\left(1 + \frac{r_{local} - r_{NP} - d_{curv} - d_{steric}}{r_{local}}\right) & s_3 \le s < s_4 \\ 0 & s \ge s_4 \end{cases}. \tag{S36}$$

Note that the orientation of the normal vector $\mathbf{n}$ is defined such that the sign of membrane curvature matches the convention in literature; that is, an inwardly curved membrane has positive mean curvature.

Plasma membrane curvature computed using Equation (S36) and the resulting FAK phosphorylation rate dictated by Equation 2 in the main text are shown in Fig S4C.

S5 Analysis of prescribed nuclear deformations

We now consider a prescribed deformation field on the nucleus. As in the case of the plasma membrane above, we formulate empirical relationships that produce shapes matching well with experiments (cf. Fig 3) rather than derive these shapes using mechanical arguments. For simplicity, we assume the nucleus adopts a reference geometry of an oblate spheroid, dictated by Equation (S5):

$$\frac{r^2}{a_{nuc}^2} + \frac{(Z - z_{nuc})^2}{b_{nuc}^2} = 1. \tag{S37}$$

For clarity, we write the reference (i.e., non-deformed) z-coordinate as Z. We assume that all deformations occur in the z-direction, so reference and deformed coordinates are the same in the x and y (and therefore, r) directions (Figure S5A). We allow a gap between the plasma membrane and the nuclear envelope, $\Delta z_{PM,NE}$, fixed 0.2 µm here. The z-component of the nuclear deformation induced by a nanopillar of radius r_{NP} and height h_{NP} located at (x_{NP}, y_{NP}) is then:

$$u_z(x,y,Z) = \begin{cases} \Delta Z_{NP} & \Delta r_{NP} \leq 0 \quad \text{and} \quad \Delta Z_{NP} \geq 0 \text{ (directly above nanopillar)} \\ \Delta Z_{NP} \exp\left(-\frac{\Delta r_{NP}^2}{\sigma^2}\right) & \Delta r_{NP} > 0 \quad \text{and} \quad \Delta Z_{NP} \geq 0 \text{ (between nanopillars)} \\ 0 & \Delta Z_{NP} < 0 \text{ (upper half of the nucleus)} \end{cases}, \tag{S38}$$

where $r_{local} = \sqrt{(x - x_{NP})^2 + (y - y_{NP})^2}$, $\Delta Z_{NP} = h_{NP} + \Delta z_{PM,NE} - Z$, and $\Delta r_{NP} = r_{local} - (r_{NP} + 2d_{steric})$. For this deformation field, the nucleus is locally flat directly above the nanopillar and within $2d_{steric}$ of the edge of the nanopillar and relaxes with a Gaussian profile away from the nanopillar as shown in Figure S5B. σ determines the width of the Gaussian profile and is fixed to 0.2 µm here. All coordinates are labeled in Figure S5C.

The local strain is characterized by the deformation gradient tensor, which is expressed in matrix form in this case as:

$$\mathbf{F} = \begin{bmatrix} 1 & 0 & 0 \\ 0 & 1 & 0 \\ u_{z,x} & u_{z,y} & 1 + u_{z,Z} \end{bmatrix}, \tag{S39}$$

where all commas in subscripts denote partial derivatives (i.e., $f_{,x} \equiv \frac{\partial f}{\partial x}$). With $\Delta x_{NP} = x - x_{NP}$ and $\Delta y_{NP} = y - y_{NP}$ expressions for each partial derivative in Equation (S39) are given by:

$$u_{z,x}(x,y,z) = \begin{cases} 0 & \Delta r_{NP} \leq 0 \quad \text{and} \quad \Delta Z_{NP} \geq 0 \\ -2\frac{\Delta Z_{NP} \Delta r_{NP} \Delta x_{NP}}{r_{local}\sigma^2} \exp\left(-\frac{\Delta r_{NP}^2}{\sigma^2}\right) & \Delta r_{NP} > 0 \quad \text{and} \quad \Delta Z_{NP} \geq 0 \\ 0 & \Delta Z_{NP} < 0 \end{cases}, \tag{S40}$$

$$u_{z,y}(x,y,z) = \begin{cases} 0 & \Delta r_{NP} \leq 0 \quad \text{and} \quad \Delta Z_{NP} \geq 0 \\ -2\frac{\Delta Z_{NP} \Delta r_{NP} \Delta y_{NP}}{r_{local}\sigma^2} \exp\left(-\frac{\Delta r_{NP}^2}{\sigma^2}\right) & \Delta r_{NP} > 0 \quad \text{and} \quad \Delta Z_{NP} \geq 0 \\ 0 & \Delta Z_{NP} < 0 \end{cases}, \tag{S41}$$

$$u_{z,Z}(x,y,z) = \begin{cases} -1 & \Delta r_{NP} \leq 0 \quad \text{and} \quad \Delta Z_{NP} \geq 0 \\ -\exp\left(-\frac{\Delta r_{NP}^2}{\sigma^2}\right) & \Delta r_{NP} > 0 \quad \text{and} \quad \Delta Z_{NP} \geq 0 \\ 0 & \Delta Z_{NP} < 0 \end{cases}. \tag{S42}$$

The area stretch ratio (termed α in the main text) is given by the kinematic relationship [11]:

$$\alpha = \frac{A}{A_0} = \det(\mathbf{F}) \left| \mathbf{n} \cdot \mathbf{F^{-1}} \right| \tag{S43}$$

$$= (1 + u_{z,Z}) \left| \begin{bmatrix} n_x \\ n_y \\ n_z \end{bmatrix} \cdot \begin{bmatrix} 1 & 0 & 0 \\ 0 & 1 & 0 \\ -\frac{u_{z,x}}{1+u_{z,Z}} & -\frac{u_{z,y}}{1+u_{z,Z}} & \frac{1}{1+u_{z,z}} \end{bmatrix} \right| \tag{S44}$$

$$= (1 + u_{z,Z}) \sqrt{\left(n_x - \frac{n_z u_{z,x}}{1 + u_{z,Z}}\right)^2 + \left(n_y - \frac{n_z u_{z,y}}{1 + u_{z,Z}}\right)^2 + \left(\frac{n_z}{1 + u_{z,Z}}\right)^2}, \tag{S45}$$

where $\mathbf{n} = [n_x, n_y, n_z]$ is the normal vector of the reference surface:

$$\mathbf{n} = \frac{a_{nuc}^2 b_{nuc}^2}{\sqrt{(x^2 + y^2) b_{nuc}^4 + (Z - z_{nuc})^2 a_{nuc}^4}} \left[\frac{x}{a_{nuc}^2}, \frac{y}{a_{nuc}^2}, \frac{Z - z_{nuc}}{b_{nuc}^2} \right]. \tag{S46}$$

Finally, substituting the above expressions yields:

$$\alpha = \begin{cases} n_z & \Delta r_{NP} \leq 0,\ \Delta Z_{NP} \geq 0 \\ (1 - \sigma_1) \sqrt{\left(n_x + \frac{2 n_z \Delta x_{NP} \sigma_1 \sigma_2}{1 - \sigma_1}\right)^2 + \left(n_y + \frac{2 n_z \Delta y_{NP} \sigma_1 \sigma_2}{1 - \sigma_1}\right)^2 + \left(\frac{n_z}{1 - \sigma_1}\right)^2} & \Delta r_{NP} > 0,\ \Delta Z_{NP} \geq 0\ , \\ 1 & \Delta Z_{NP} < 0 \end{cases} \tag{S47}$$

$$\text{where } \sigma_1 = \exp\left(-\frac{\Delta r_{NP}^2}{\sigma^2}\right) \quad \text{and} \quad \sigma_2 = \frac{\Delta Z_{NP} \Delta r_{NP}}{r_{local} \sigma^2}.$$

The curvature of the membrane directly above a nanopillar is identically zero. To compute the curvature of the nuclear membrane elsewhere when $\Delta Z_{NP} \geq 0$, we represent it as a Monge patch [12]:

$$z(x, y) = Z + \Delta Z_{NP} \exp\left(-\frac{\Delta r_{NP}^2}{\sigma^2}\right) \tag{S48}$$

For convenience, we first define the partial derivatives associated with the reference ellipsoidal surface:

$$\begin{aligned} Z_{,x} &= \frac{b_{nuc} x}{a_{nuc}^2 \alpha} \\ Z_{,y} &= \frac{b_{nuc} y}{a_{nuc}^2 \alpha} \\ Z_{,xy} &= \frac{b_{nuc} xy}{a_{nuc}^4 \alpha^3} \\ Z_{,xx} &= \frac{b_{nuc} x^2}{a_{nuc}^4 \alpha^3} + \frac{b_{nuc}}{a_{nuc}^2 \alpha} \\ Z_{,yy} &= \frac{b_{nuc} y^2}{a_{nuc}^4 \alpha^3} + \frac{b_{nuc}}{a_{nuc}^2 \alpha}, \\ \text{where} \quad \alpha &= \sqrt{1 - \frac{x^2 + y^2}{a_{nuc}^2}}. \end{aligned} \tag{S49}$$

The mean curvature is then given explicitly by:

$$H_{NE}(x,y) = -\frac{(1+z_{,y}^2)z_{,xx} - 2z_{,x}z_{,y}z_{,xy} + (1+z_{,x}^2)z_{,yy}}{2(1+z_{,x}^2+z_{,y}^2)^{3/2}}, \tag{S50}$$

where

$$\begin{aligned}
z_{,x} &= Z_{,x}(1-\sigma_1) - 2\sigma_1\sigma_2\Delta x_{NP} \\
z_{,y} &= Z_{,y}(1-\sigma_1) - 2\sigma_1\sigma_2\Delta y_{NP} \\
z_{,xy} &= Z_{,xy}(1-\sigma_1) + 2\sigma_1\sigma_2\Delta x_{NP}\Delta y_{NP}\left(\frac{Z_{,x}}{\Delta Z_{NP}\Delta x_{NP}} + \frac{Z_{,y}}{\Delta Z_{NP}\Delta y_{NP}} + \frac{2\Delta r_{NP}}{\sigma^2 r_{local}} + \frac{1}{r_{local}^2} - \frac{1}{r_{local}\Delta r_{NP}}\right) \\
z_{,xx} &= Z_{,xx}(1-\sigma_1) + 2\sigma_1\sigma_2\Delta x_{NP}^2\left(\frac{2Z_{,x}}{\Delta Z_{NP}\Delta x_{NP}} + \frac{2\Delta r_{NP}}{\sigma^2 r_{local}} + \frac{1}{r_{local}^2} - \frac{1}{r_{local}\Delta r_{NP}} - \frac{1}{\Delta x_{NP}^2}\right) \\
z_{,yy} &= Z_{,yy}(1-\sigma_1) + 2\sigma_1\sigma_2\Delta y_{NP}^2\left(\frac{Z_{,y}}{\Delta Z_{NP}\Delta y_{NP}} + \frac{2\Delta r_{NP}}{\sigma^2 r_{local}} + \frac{1}{r_{local}^2} - \frac{1}{r_{local}\Delta r_{NP}} - \frac{1}{\Delta y_{NP}^2}\right).
\end{aligned}$$

The above equations consider deformation ascribed to a single nanopillar acting on the nuclear envelope. To ensure continuity of membrane deformations in response to multiple nanopillars, we simply consider the summation of effects at a given point, provided it is not directly above a nanopillar:

$$u_z \leftarrow \sum_{n=1}^{N} \begin{cases} \Delta z_{NP} \exp\left(-\frac{(r_{local,n}-(r_{NP}+2d_{steric}))^2}{\sigma^2}\right) & \Delta Z_{NP} \geq 0 \\ 0 & \Delta Z_{NP} < 0 \end{cases} \tag{S51}$$

where N is the total number of nanopillars under consideration and $r_{local,n} = \sqrt{(x-x_{NP,n})^2 + (y-y_{NP,n})^2}$ is the distance from nanopillar n. Generally speaking, because $\sigma = 0.2$ µm, which is less than the spacing between nanopillars, the deformations are mainly attributed to the nanopillar closest to a given point on the nuclear envelope. If the point under consideration is in the upper region of the nuclear envelope (i.e., $Z_{NP} < 0$), then the curvature is given by setting $\sigma_1 = \sigma_2 = 0$ in Equation (S50).

To ensure conservation of volume for the nucleus, we adjust a_{nuc} in Equation (S37) such that the deviation in volume is less than 1% after applying the indentation described above.

Supporting Figures and Movies

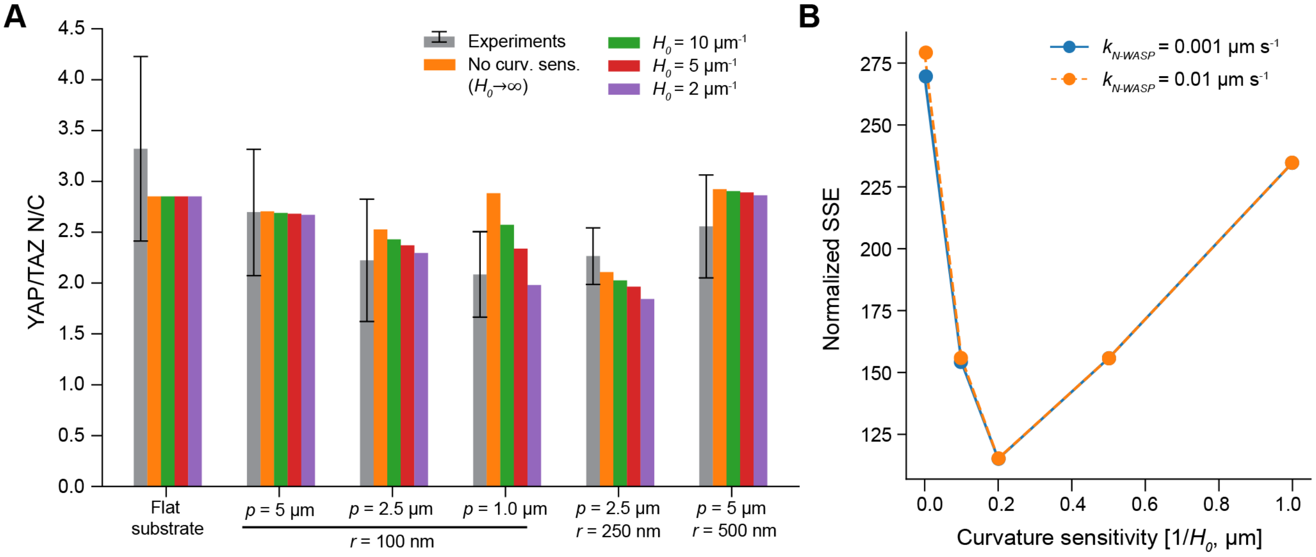

Figure S1: Determination of best-fit FAK curvature sensitivity. A) YAP/TAZ N/C in experiments (grey bars: mean ± standard deviation) and in simulations (spatially averaged at steady-state) with different levels of curvature sensitivity. B) Normalized SSE (Eq S9) as a function of curvature sensitivity ($1/H_0$) for two different rates of N-WASP-mediated actin assembly at regions of high PM curvature.

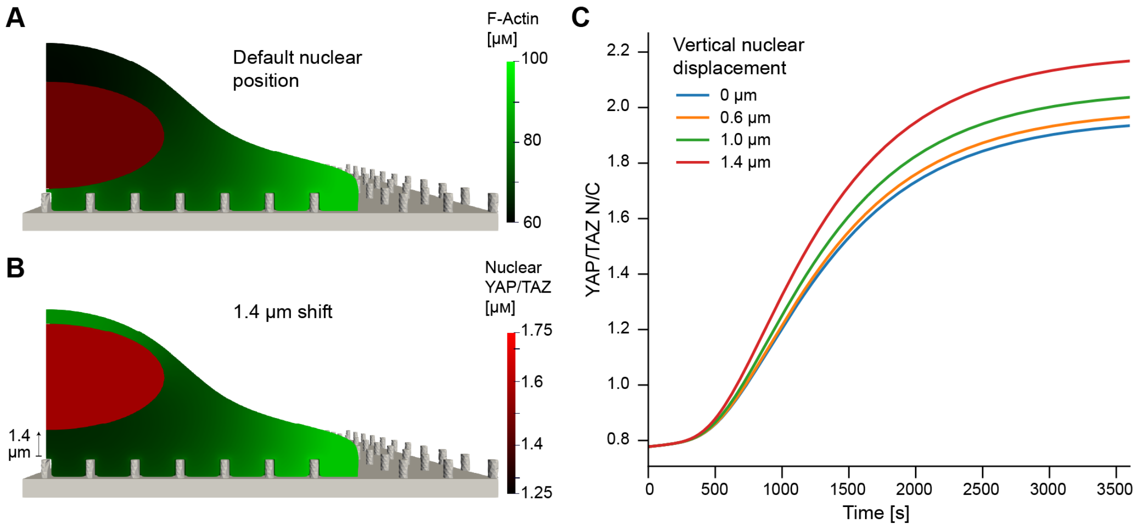

Figure S2: Decreasing the gap between the nucleus and upper plasma membrane enhances YAP/TAZ nuclear entry. A-B) Contrast in nuclear YAP/TAZ and F-actin distributions between the default nuclear position (A) and a nucleus shifted up by 1.4 µm (B) at steady-state (t = 10,000 s). C) YAP/TAZ N/C dynamics for different nuclear locations, showing increased nuclear accumulation for larger vertical shifts. All simulations in this figure were conducted for a substrate with r_{NP} = 250 nm, p_{NP} = 2.5 µm, and h_{NP} = 1 µm.

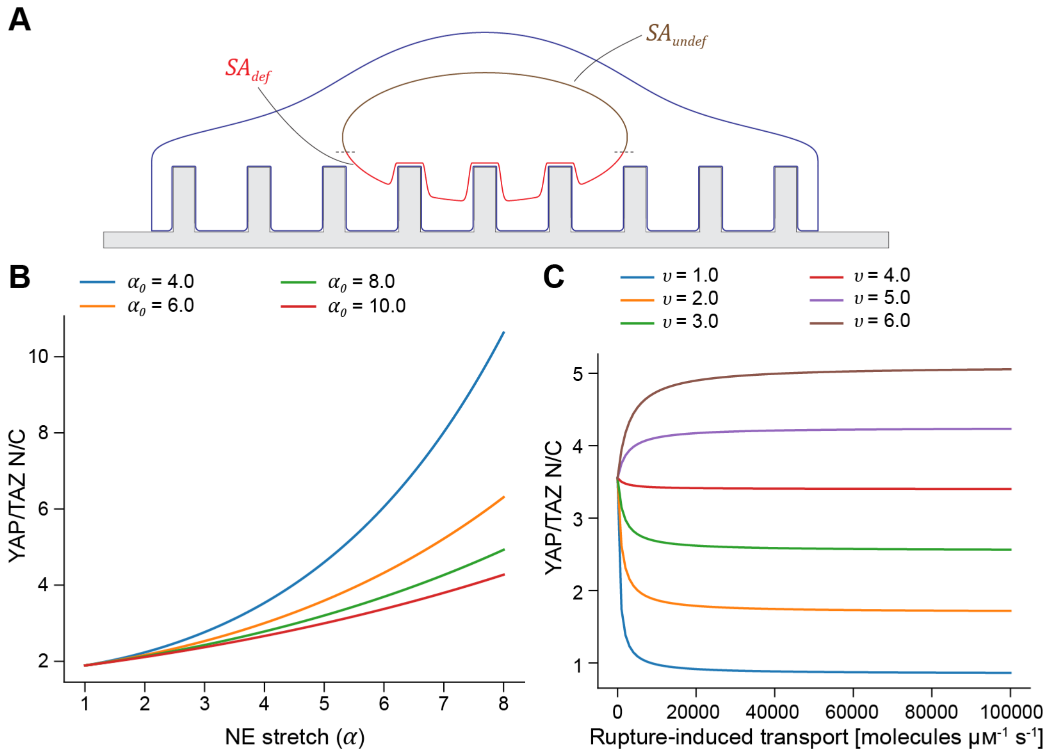

Figure S3: Well-mixed model predictions for YAP/TAZ N/C. A) Schematic showing two regions of the nuclear envelope (deformed and undeformed) considered in the well-mixed model. B) Predicted stretch-dependent effects on YAP/TAZ transport through NPCs using Eq 5 and $SA_{def} \approx \alpha SA_0$. Based on values in spatial simulations of indented nuclei, we use $\phi_{free} = 0.85$, $[NPC_A]_{def} = 0.3$, $[NPC_A]_{undef} = 0.1$, $SA_0 = 5$ μm^2, and $SA_{undef} = 40$ μm^2. C) Predicted NER-dependent effects on YAP/TAZ transport through NPCs using Eq 5. We use the same parameters as in panel B, but with $\alpha = 4.5$ and $\alpha_0 = 5$; the x-axis corresponds to $SA_{NER} k_{rupture}$.

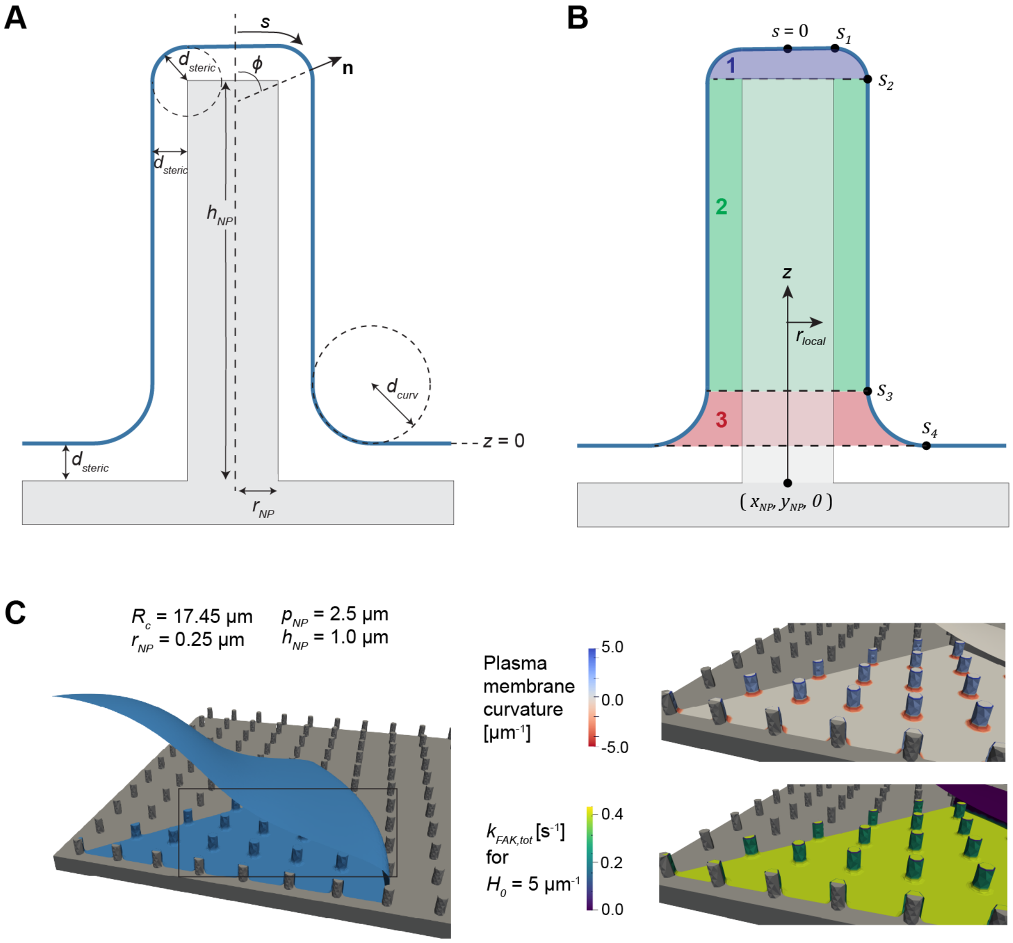

Figure S4: Schematics of plasma membrane deformation on nanopillar substrates. A) Cross-section of the PM (blue) near the surface of a cylindrical nanopillar. Relevant parameters required for calculation of the PM curvature (Eq S36) are defined graphically. This section of the geometry is axisymmetric with respect to the central axis of the nanopillar (dashed line). B) PM cross section with volumetric regions 1, 2, and 3 corresponding to those in Equation (S4) and arc length coordinates (s_1, s_2, s_3, and s_4) corresponding to those in Equations (S32) to (S36). C) A sample PM geometry (left, blue surface) on a substrate with 250 nm radius nanopillars ($p_{NP} = 2.5$ μm, $h_{NP} = 1.0$ μm), along with the resulting PM curvature (top right) and the local rate of curvature-dependent FAK phosphorylation when $H_0 = 5\text{μm}^{-1}$ (lower right).

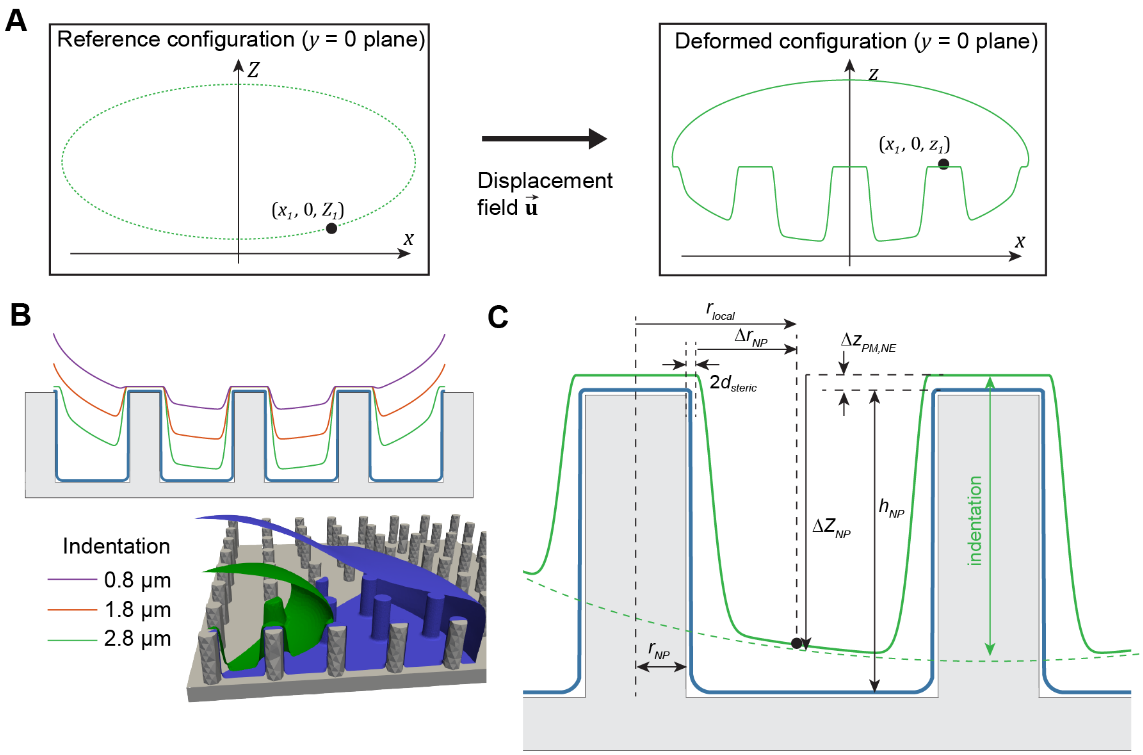

Figure S5: Schematics of nuclear deformation on nanopillar substrates. A) Summary of reference coordinates (x, y, Z) (upper) vs. deformed coordinates (x, y, z) (lower) over the $y = 0$ cross section of the nuclear envelope. B) A cross-section $(y = 0)$ through the center of the cell showing prescribed deformations of the NE for different values of nuclear indentation (upper), with the 3D surface mesh associated with a deformation of 2.8 µm (lower). PM configuration is plotted in blue. C) Magnified schematic with local coordinates (r_{local}, Δr_{NP}, and ΔZ_{NP}) defined with respect to the left nanopillar. $r_{local} = \sqrt{(x - x_{NP})^2 + (y - y_{NP})^2}$, $\Delta Z_{NP} = h_{NP} + \Delta z_{PM,NE} - Z$, and $\Delta r_{NP} = r_{local} - (r_{NP} + 2d_{steric})$.

Movie 1: Fast time-scale dynamics of F-actin in a cell on a substrate with 500 nm radius nanopillars (5.0 μm spacing, 1.0 μm height).

Movie 2: Fast time-scale dynamics of F-actin in a cell on a substrate with 250 nm radius nanopillars (2.5 μm spacing, 1.0 μm height).

Movie 3: Fast time-scale dynamics of F-actin in a cell on a substrate with 100 nm radius nanopillars (2.5 μm spacing, 1.0 μm height).

Movie 4: Spatial dynamics of cytosolic F-actin and nuclear YAP/TAZ in a cell on a flat substrate (substrate stiffness = 10 GPa).

Movie 5: Spatial dynamics of cytosolic F-actin and nuclear YAP/TAZ in a cell on a substrate with 100 nm radius nanopillars (2.5 μm spacing, 1.0 μm height).

Movie 6: Spatial dynamics of cytosolic F-actin and nuclear YAP/TAZ in a cell on a substrate with 100 nm radius nanopillars (5.0 μm spacing, 1.0 μm height).

Movie 7: Spatial dynamics of cytosolic F-actin and nuclear YAP/TAZ in a cell on a substrate with 500 nm radius nanopillars (3.5 μm spacing, 3.0 μm height) with no nuclear indentation.

Movie 8: Spatial dynamics of cytosolic F-actin and nuclear YAP/TAZ in a cell on a substrate with 500 nm radius nanopillars (3.5 μm spacing, 3.0 μm height) with 0.8 μm nuclear indentation.

Movie 9: Spatial dynamics of cytosolic F-actin and nuclear YAP/TAZ in a cell on a substrate with 500 nm radius nanopillars (3.5 μm spacing, 3.0 μm height) with 1.8 μm nuclear indentation.

Movie 10: Spatial dynamics of cytosolic F-actin and nuclear YAP/TAZ in a cell on a substrate with 500 nm radius nanopillars (3.5 μm spacing, 3.0 μm height) with 2.8 μm nuclear indentation.

Movie 11: Spatial dynamics of cytosolic and nuclear phosphorylated YAP/TAZ following nuclear envelope rupture (pore radius = 200 nm) in a cell with 2.8 μm nuclear indentation, assuming $\upsilon = 5$. The boundary of the nucleus is shaded to indicate NE permeability.

Movie 12: Spatial dynamics of cytosolic and nuclear phosphorylated YAP/TAZ following nuclear envelope rupture (pore radius = 200 nm) in a cell with 2.8 μm nuclear indentation, assuming $\upsilon = 1$. The boundary of the nucleus is shaded to indicate NE permeability.

References

1. Geuzaine, C. & Remacle, J.-F. Gmsh: A 3-D Finite Element Mesh Generator with Built-in Pre- and Post-Processing Facilities: THE GMSH PAPER. *International Journal for Numerical Methods in Engineering* **79,** 1309–1331. ISSN: 00295981 (Sept. 2009).
2. Scott, K. E., Fraley, S. I. & Rangamani, P. A Spatial Model of YAP/TAZ Signaling Reveals How Stiffness, Dimensionality, and Shape Contribute to Emergent Outcomes. *Proceedings of the National Academy of Sciences* **118,** e2021571118. ISSN: 0027-8424, 1091-6490 (May 2021).
3. Meurer, A. *et al.* SymPy: Symbolic Computing in Python. *PeerJ Computer Science* **3,** e103. ISSN: 2376-5992 (Jan. 2017).
4. Beck, M. *et al.* The Quantitative Proteome of a Human Cell Line. *Molecular Systems Biology* **7,** 549. ISSN: 1744-4292 (Nov. 2011).

5. Koch, B. *et al.* Confinement and Deformation of Single Cells and Their Nuclei Inside Size-Adapted Microtubes. *Advanced healthcare materials* **3,** 1753–1758. ISSN: 2192-2640 (Nov. 2014).
6. Francis, E. A. *et al.* Spatial Modeling Algorithms for Reactions and Transport in Biological Cells. *Nature Computational Science,* 1–14. ISSN: 2662-8457 (Dec. 2024).
7. Singh, A. P. *et al.* 3D Protein Dynamics in the Cell Nucleus. *Biophysical Journal* **112,** 133–142. ISSN: 0006-3495, 1542-0086 (Jan. 2017).
8. Zhu, K. *et al.* Membrane Curvature Catalyzes Actin Nucleation through Nano-Scale Condensation of N-WASP-FBP17. bioRxiv (preprint): 10.1101/2024.04.25.591054, 2024.04.25.591054 (Apr. 2024).
9. Lou, H.-Y. *et al.* Membrane Curvature Underlies Actin Reorganization in Response to Nanoscale Surface Topography. *Proceedings of the National Academy of Sciences* **116,** 23143–23151 (Nov. 2019).
10. Li, X. *et al.* Nanoscale Surface Topography Reduces Focal Adhesions and Cell Stiffness by Enhancing Integrin Endocytosis. *Nano letters* **21,** 8518–8526. ISSN: 1530-6984 (Oct. 2021).
11. Taber, L. A. *Nonlinear Theory of Elasticity: Applications in Biomechanics* ISBN: 978-981-238-735-6 978-981-279-422-2 (WORLD SCIENTIFIC, Feb. 2004).
12. Weisstein, E. W. *Monge Patch* https://mathworld.wolfram.com/MongePatch.html. Text.
